# Mobilizing domestic support for international vaccine solidarity – recommendations for health crisis communication

**DOI:** 10.1038/s41541-023-00625-x

**Published:** 2023-02-28

**Authors:** Dirk Leuffen, Pascal Mohamed Mounchid, Max Heermann, Sebastian Koos

**Affiliations:** 1 Department of Politics and Public Administration, Cluster of Excellence “Politics of Inequality”, University of Konstanz, Postfach 83, Universitätsstrasse 10, 78457 Konstanz, Germany; 2 Department of Politics and Public Administration, University of Konstanz, Postfach 83, Universitätsstrasse 10, 78457 Konstanz, Germany; 3 Department of Sociology, Cluster of Excellence “Politics of Inequality”, University of Konstanz, Universitätsstrasse 10, 78457 Konstanz, Germany

**Keywords:** Public health, Drug regulation

## Introduction

Throughout the COVID-19 pandemic, global inequality in the access to vaccines has been highlighted as a moral injustice and a medical danger. Medical experts agree that global vaccine inequity risks the emergence of vaccine-resistant virus mutations, which threaten to undo the progress made in countries with high vaccination rates^1,2^. At the same time, shortcomings of international solidarity are recurrently deplored. For instance, in 2021, World Health Organization (WHO) Director Tedros Adhanom Ghebreyesus accused high- and upper-middle income countries of ‘vaccine nationalism’, severely putting at risk lower-income countries, which at that time had only received about 0.3 per cent of all global vaccinations (*New York Times* 2021-04-22). In the first half of 2021, the European Union (EU), which had been one of the initial driving forces of COVAX, the vaccine pillar of the Access to COVID-19 Tools (ACT) Accelerator, clearly lagged behind its own ambitions, leading observers to criticize its faltered leadership^3^. Thus, despite the recognition that the ‘extensive immunization against COVID‐19 [is] a global public good’ (G20 Summit Declaration of November 2020), and that ‘no one is safe, until everyone is safe’, shortcomings in solidarity became apparent^4–7^.

In this commentary we argue and empirically show that policy-makers in donor countries can foster public support for international vaccine solidarity by providing their citizens with the right mix of information about citizens’ long-term self-interest in sharing vaccines, as well as information on the medical situation in potential recipient countries. We show that the effects on undecided voters are particularly strong. Whereas extant research has informed about which types of citizens support international solidarity^8^, we so far know less about how to mobilize reluctant publics – and this is a much regretted shortcoming, given the need for international cooperation in diverse fields^9,10^.

We present a catalogue of five evidence-based recommendations for crisis communication in support of global health solidarity. The recommendations are derived from survey experimental research, conducted in Germany during COVID-19. The survey was in the field in May 2021, at a time when, according to the Robert Koch-Institut, Germany’s public health institute, about 37 per cent of citizens had received their first, and only about 12 per cent their second vaccine shot. The experiments are anchored in social science theory and research^11^. The results highlight, first, that support for vaccine solidarity increases when citizens are well-informed about the medical logic of sharing vaccines. Informing citizens about the fact that a global vaccine rollout reduces the risk of future vaccine-resistant virus mutations – and is thus in their own interest – increases their support for international vaccine solidarity. Second, politicians and experts must point out medium- and long-term benefits of vaccine solidarity, to escape the trap of short-termism. Third, citizens are sensitive with respect to need: in line with expectations from evolutionary psychology and moral philosophy, solidarity increases with the perceived degree of need. Therefore, communicating the severity of what is at stake remains important. Fourth, fault attribution matters. If potential recipients cannot be blamed for their adverse situation, they are perceived to be more deserving of aid. Fifth, advocates of solidarity should highlight the ties between donors and recipients – homophily and expected reciprocity make people more generous.

## Domestic constraints to international health solidarity

Conventionally, failures to achieve international cooperation and solidarity are explained by collective action problems and a lack of credible enforcement mechanisms. More recently, the role of domestic politics in constraining international cooperation has received growing attention. In times of populism and politicized foreign affairs, policy-makers find it increasingly difficult to justify expenses directed at international aid and multilateralist action^12,13^. This challenge is particularly troubling in democracies: politicians are oftentimes torn between following the advice of experts and, at the same time, honouring responsiveness vis-à-vis the public.

One reason for the shortcomings in the implementation of the global rollout of vaccines are electoral incentive structures, which favour domestic demand in donor states at the expense of protecting the most vulnerable persons around the globe. While political elites may appreciate the merits of multilateralism and trust their international partners, they still have to justify their support of international cooperation and solidarity back at home. And here populism can be a true hindrance, as highlighted by US President Donald Trump’s decision to turn against the World Health Organization (WHO) in the early days of the pandemic^14^.

## Analyzing support for the international rollout of vaccines

To better understand how perceptions of recipient characteristics and need situations affect donor state citizens’ support of international solidarity, we conducted a series of survey experiments^15^ in Germany. The empirical information reported in this article comes from the May 2021 wave, in which we directly address support for vaccine solidarity.

The modules were part of the COVID-19 and Inequality Survey Program, financed by the Cluster of Excellence for The Politics of Inequality, Konstanz, Germany, and administered by the survey firm Respondi (today Bilendi); the study was approved by the ethics committee of the University of Konstanz and all subjects of the online access panel provided written informed consent. The survey employed quota sampling based on age, gender, level of education and region (federal unit) and included people aged 18 and over and residing in Germany. Before data cleaning, the sample size was 4027 persons, afterwards 4019. Supplementary Table 1, compares the quotas from the Federal Statistical Office and the sample drawn, revealing individual over- and under-representations. Individual sample weights were therefore added to the analysis to compensate for these deficits. Balance tests (two sample *t*-test) were carried out to check the necessary randomised distribution of the level expressions.

In May 2021, Germany experienced its third COVID infection wave and the national vaccination campaign was slowly extended from high-risk groups to wider sections of the population. Germany is an important case for studying public support for vaccine donations, as it is the most populous and economically most powerful EU member state. Moreover, the country has taken a leading role in the EU’s joint response to the pandemic during its presidency of the Council of the EU in the second half of 2020.

We combined a randomized information treatment with a factorial survey (vignette) experiment. The information treatment explained the medical logic behind vaccine solidarity: global inequality in access to vaccines risks the emergence of new, potentially vaccine-resistant virus variants endangering the progress achieved in rich countries with successful vaccination campaigns^16^. The information treatment thus emphasises that vaccine solidarity is not merely a form of charity but serves the interests of the donor country. Kobayashi et al.^17^ propose a similar mechanism, underlining that perceived sociotropic benefits can enhance citizens’ support for development aid.

The vignettes described hypothetical recipient countries in need of vaccines. The respondents were then asked, whether they would support sending vaccines to these countries, with different degrees of support being measured on a 7-point Likert scale from 1 (‘I do not support at all’) to 7 (‘I fully support’). The vignette attributes were developed to reflect state-of-the-art social science theories, relating mostly to redistributive preferences, as derived from the welfare state and development aid literatures^11^.

Following evolutionary psychology and moral philosophy, we expect solidarity to rise with the levels of need. In the vignettes, we operationalize medical need by providing information on an acute shortage in intensive-care units (ICUs), as compared to a more moderate baseline situation. Based on insights from behavioural economics and social psychology we inspect whether “deservingness” and fault attribution matter for citizens’ support of aid programmes. In particular, we inform respondents about the pandemic management in the hypothetical recipient country, varying a swift versus slow introduction of containment measures to fight COVID-19. To account for similarity between the donor and a recipient country, we, first, vary the recipient country’s political regime type. We expected that German citizens should appreciate sending vaccines to other democracies, partly because of homophily and possibly because these countries can be expected to reciprocate aid. Finally, we also control for costs, since strong evidence stresses that with rising costs, (vaccine) solidarity decreases^18–20^. We operationalize costs by informing respondents about whether the EU is close to versus still far from herd immunity, as well as through geographic distance; in particular, we compare support for solidarity with a country neighbouring the EU and Latin-American country. Supplementary Tables 2–4 provide more detailed information on the vignette and the treatments.

## Vaccine solidarity is conditional

**Fig. 1:**
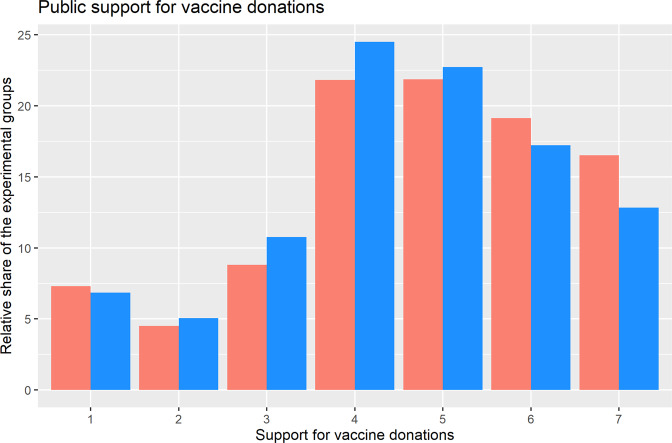
Support for vaccine sharing, separated by experimental groups (‘information treatment’); treatment group in red, control group in blue; likert scale ranging from complete objection to full support. Data collected in Germany in May 2021, *n* = 4022 (treatment group *n* = 1974, control group *n* = 2048).

How supportive are German citizens of international vaccine solidarity? On average, 55 per cent of respondents expressed their support for vaccine donations to the hypothetical recipient countries described in the vignette, indicating an overall quite high support for vaccine redistribution (mean treatment group: 4,69, standard deviation: 1,70; mean control group: 4,54, standard deviation: 1,66). Only 22 per cent expressed their opposition to vaccine sharing, and a similar percentage of respondents were undecided (23 per cent). Obviously, we cannot exclude social desirability effects, which may contribute to explaining the overall high support for solidarity. However, a visual inspection of the differences between the group which has received the information treatment (red bars in Fig. 1) and the control group (blue bars in Fig. 1) shows that the information treatment has a particularly large effect on the undecided citizens (see Fig. 1). Informing respondents about the instrumental benefits of sharing vaccines – i.e., reducing the likelihood of virus mutations – decreases the percentage of undecided respondents (category 4 on Likert-scale) by almost three percentage points, while increasing the support camp (i.e., category 6 and 7 on Likert-scale) by 5.6 percentage points.

**Fig. 2:**
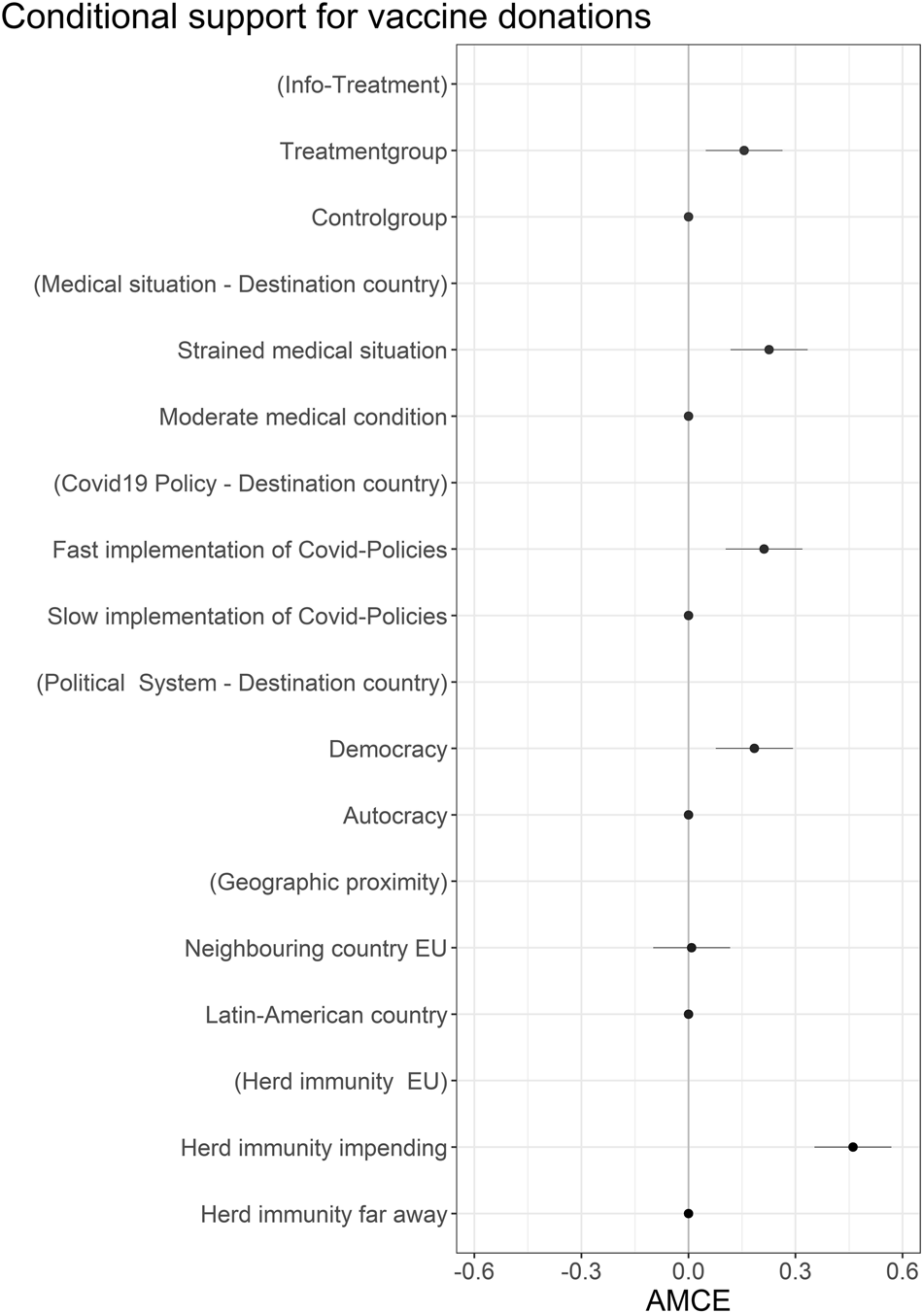
Conditional public support for international vaccine solidarity. Average marginal component effects of experimental treatments. Data collected in Germany in May 2021, *n* = 4022.

The experimental results are reported in Fig. 2. Clearly, information on the beneficial effects of sharing vaccines significantly increases support for such measures. Recoding the dependent variable in a dichotomous measure, which distinguishes between no support (1–4 on the Likert-scale = 0) and support for vaccine solidarity (5–7 on the Likert-scale = 1), allows to report the increase in the likelihood of support for each vignette dimension or factor and thus provides an intuitive understanding of effect sizes. Based on a linear probability model the information treatment increases the likelihood of support for international vaccine solidarity by almost 5 percentage points (AMCE: 0.156, 95% CI: 0.048–0.263, *p*-value: 0.004) (see model 1, Supplementary Table 5). Next, the vignette experiment underlines that citizens take medical need very seriously; need increases support by 6 percentage points (AMCE: 0,226, 95% CI: 0.118–0.334, *p*-value: 0.000). Citizens are concerned about deservingness, as they appreciate robust actions taken to fight the pandemic; a fast implementation of virus containment policies increases support by 4 percentage points (AMCE: 0.211, 95% CI: 0.104–0.319, *p*-value: 0.000). Our respondents seem to value homophily by showing greater willingness to donate to democratic, as compared to non-democratic states; political homophily increases support by 4 percentage points (AMCE: 0.184, 95% CI: 0.076–0.292, *p*-value: 0.000). Geographical distance does not seem to matter; in fact, our respondents do not display a stronger support for vaccines being sent to a neighbouring country of the EU, rather than to a Latin American country (AMCE: 0.008, 95% CI: −0.098–0,116, *p*-value: 0.871). However, as expected, costs, operationalized by lower versus higher chances of achieving herd immunity in the EU, significantly reduce support for a global sharing of vaccines; assuming herd immunity in the EU is nearly reached, increases support by almost 14 percentage points (AMCE: 0.460, 95% CI: 0.353–0.568, *p*-value: 0.000). Successful domestic vaccine campaigns in donor countries therefore contribute to public support for global solidarity; note that in this respect, focussing on surplus vaccines makes a lot of sense^21^.

Our experiments have focused on the role information, recipient characteristics and costs, adding to a well-established literature, in which individuals’ attitudes and personality characteristics, such as altruism or cosmopolitan identity make citizens more supportive of international solidarity. In line with this latter research, our analyses also confirm that self-declared altruists are more likely to support vaccine solidarity (see Supplementary Table 5).

## How to advocate vaccine solidarity

What can the supporters of global health solidarity learn from these results? Acknowledging that international solidarity is not a fast-selling policy, they must invest ‘ideational leadership’ to promote their policy preferences^22,23^. In this respect, crisis communication is an important instrument. The experimental results call for integrating moral appeals to charity with instrumental motives promoting the donors’ self-interest (Box 1). The slogan, frequently used during the COVID-19 pandemic, that ‘no one is safe, until everyone is safe’ gets it right. Despite evident expert knowledge of such mechanisms, political actors should not get tired of stressing these mechanisms time and again – citizens have many other things on their minds, and thus need to be made aware and reminded of what is at stake. Likewise, emphasizing the existential medical need of potential recipients makes citizens more compassionate and therefore more generous. Highlighting the health policy efforts of recipient states and creating ties between donors and recipients enhances public support for solidarity. Obviously, political communication must aim at simplicity – the audience must get the message – but avoid oversimplification, to not mirror populist rhetoric and thereby lose credibility. Those that are strongly against international vaccine solidarity (1 and 2 on our 7-point Likert scale) are hardly affected in their evaluation by the treatments (see Fig. 1); our research thus indicates that expert information may not convince those parts of societies that have fallen prey to ‘alternative facts’ and ‘post truth’ beliefs. However, we present evidence that expert information can positively shape the attitudes of undecided citizens. Both, moral and instrumental reasons justify support of international solidarity and contribute to successfully building public support coalitions for global health and beyond.

Box 1 Five recommendations for mobilizing public support for international vaccine solidarity

1. Mention the causal mechanisms. Do not shy away from detailing the costs of vaccine inequality and the benefits of a global vaccine rollout.
2. Escape the trap of short-termism: move the focus from immediate costs to long-term benefits.
3. Need matters! Moving beyond self-interest, citizens also value charity.
4. Avoid simplified fault attribution schemes – deservingness increases solidarity.
5. Evoking similarity between donors and recipients strengthens reciprocity-based solidarity.

## Supplementary information

Supplementary Information Files

## Data Availability

The replication data is available at 10.7910/DVN/6KJHSN, Harvard Dataverse. The data set is part of the ‘COVID-19 and Inequality - Survey Program’ of the Cluster of Excellence on ‘The Politics of Inequality’, available at 10.7802/2456.

## Code availability

The replication code is available at 10.7910/DVN/6KJHSN, Harvard Dataverse.
