# Supplementary material for: Mobilizing domestic support for international vaccine solidarity – recommendations for health crisis communication
Source: NPJ Vaccines. 2023 Feb 28;8:28. doi: 10.1038/s41541-023-00625-x (PMC9972312; doi:10.1038/s41541-023-00625-x)
Supplement: Supplementary file 1 — Supplementary Information Files [file 41541_2023_625_MOESM1_ESM.pdf]

**Supplementary Information Files**

**Supplement to: Leuffen D., Mounchid P. M., Heermann M., Koos S.**

**Mobilizing Domestic Support for International Vaccine Solidarity – Recommendations for Health Crisis Communication**

**Supplementary Tables**

1. Supplementary Table 1: Sample composition compared to population census data
2. Supplementary Table 2: Experimental set-up
3. Supplementary Table 3: Information experiment
4. Supplementary Table 4: Attributes of the vignette experiment
5. Supplementary Table 5: Public support for vaccine solidarity: linear probability models

## Supplementary Tables

| | Sample | Population (census) |
|---|---|---|
| Highest educational level attained: | | |
| Lower education | 37.4% | 35.8 % |
| Secondary schooling | 30.2% | 30.5 % |
| Higher education | 32.4% | 33.6 % |
| Female | 49.0% | 50.1 % |
| Age: | | |
| 18-39 years | 27.0% | 31.6 % |
| 40-59 years | 39.5% | 35.2 % |
| 60+ years | 33.5% | 33.2 % |
| East | 29.0% | 19.7 % |

*Note: East Germany was deliberately oversampled in the survey.*

### Supplementary Table 1: Sample composition (unweighted) compared to population census data

*Now we are interested in your attitude towards a global redistribution of vaccines.*

*The European Union is one of the most important production sites for Corona virus vaccines. Therefore, it is heavily debated, whether the EU should send EU vaccines to states, which are not EU member states, and which do not possess vaccine production sites of their own.*

**[According to experts, global vaccination efforts must be enacted to contain the Corona virus pandemic. The faster global vaccination efforts proceed, the smaller the risk of the emergence of vaccine resistant virus mutations.]**

*Imagine a country, demanding vaccine doses from the EU in the summer of 2021. Would you support allocating vaccine doses to this country?*

The **[democratically governed | autocratically governed] [EU neighbour state | Latin American state]** was hit hard by the pandemic, **[despite its government having enacted rapid measures to contain the virus | not least because its government only hesitantly enacted measures to contain the virus]**. **[Its intensive care units have clearly been overburdened | Its intensive care units have not yet been overburdened.]** The EU **[is close to | is still far from]** reaching herd immunity. Would you support the allocation of vaccine doses to this country?

*Answer category ranges from "I support this not at all" to "I support this very strongly" (7-point Likert scale).*

### Supplementary Table 2: Experimental set-up

| Information Treatment | |
|---|---|
| **Treatment Group** | **Control Group** |
| The European Union is one of the most important production sites for Corona virus vaccines. Therefore, it is heavily debated, whether the EU should send EU vaccines to states, which are not EU member states, and which do not possess vaccine production sites of their own.<br><br>According to experts, global vaccination efforts must be enacted to contain the Corona virus pandemic. The faster global vaccination efforts proceed, the smaller the risk of the emergence of vaccine resistant virus mutations. | The European Union is one of the most important production sites for Corona virus vaccines. Therefore, it is heavily debated, whether the EU should send EU vaccines to states, which are not EU member states, and which do not possess vaccine production sites of their own. |

## Supplementary Table 3: Information experiment

| Vignette | |
|---|---|
| **Attributes** | **Levels** |
| Need: Medical situation in destination country | 1. Strained medical situation<br>2. Moderate medical condition |
| Deservingness: Implementation of COVID-19 policies in destination country | 1. Fast implementation<br>2. Slow implementation |
| Homophily: Political System in destination country | 1. Democracy<br>2. Autocracy |
| Geographic proximity of destination country to donor country | 1. Latin-American country<br>2. Neighbouring country EU |
| Costs: Herd immunity in EU | 1. Herd immunity far away<br>2. Herd immunity impending |

## Supplementary Table 4: Attributes of the vignette experiment

| | Dependent variable: | |
|---|---|---|
| | Support for vaccine solidarity (dichotomized) | |
| | (1) | (2) |
| Information treatment | 0.047*** | 0.036** |
| | (0.015) | (0.015) |
| Strained medical situation | 0.064*** | 0.063*** |
| | (0.016) | (0.015) |
| Fast implementation of Covid policies | 0.043*** | 0.051*** |
| | (0.015) | (0.015) |
| Democracy | 0.039** | 0.048*** |
| | (0.016) | (0.015) |
| Neighbouring country EU | 0.018 | 0.018 |
| | (0.015) | (0.015) |
| Herd immunity impending | 0.135*** | 0.135*** |
| | (0.015) | (0.015) |
| Age | | 0.002*** |
| | | (0.0005) |
| Education level high (ref. low) | | 0.126*** |
| | | (0.019) |
| Education level middle (ref. low) | | 0.047*** |
| | | (0.018) |
| Female | | -0.030** |
| | | (0.015) |
| Altruism | | 0.090*** |
| | | (0.005) |
| Constant | 0.378*** | -0.217*** |
| | (0.020) | (0.042) |
| Observations | 4,022 | 4,012 |
| $R^2$ | 0.028 | 0.126 |
| Adjusted $R^2$ | 0.027 | 0.123 |
| Residual Std. Error | 0.512 (df = 4015) | 0.486 (df = 4000) |
| F Statistic | 19.525*** (df = 6; 4015) | 52.342*** (df = 11; 4000) |
| *Note:* | | *p**p***p<0.01 |

**Supplementary Table 5: Public support for vaccine solidarity: linear probability models**
